# Cost and Utilization of Ambulance Services Across the United States

**DOI:** 10.3390/healthcare14081073

**Published:** 2026-04-17

**Authors:** Vanessa A. Moore, Austin Watkins, Michael Ting, Ben Seibert, Justin Dvorak, Katie Keyser, Nirmal Choradia, Ryan D. Nipp

**Affiliations:** 1 College of Medicine, University of Oklahoma Health Sciences Center, Oklahoma City, OK 73117, USA; austin-watkins@ouhsc.edu (A.W.); michael-ting@ouhsc.edu (M.T.); 2 Department of Medicine, University of Oklahoma Health Sciences Center, Oklahoma City, OK 73117, USA; benjamin-seibert@ouhsc.edu; 3 Department of Biostatistics & Epidemiology, Hudson College of Public Health, University of Oklahoma Health Sciences Center, Oklahoma City, OK 73117, USA; justin-dvorak@ouhsc.edu; 4 Division of Hematology & Oncology, Department of Medicine, Stephenson Cancer Center, University of Oklahoma Health Campus, Oklahoma City, OK 73117, USA; katie-keyser@ouhsc.edu (K.K.); nirmal-choradia@ouhsc.edu (N.C.); ryan-nipp@ouhsc.edu (R.D.N.)

**Keywords:** ambulance, ambulance services, cost, expense

## Abstract

**Highlights:**

**What are the main findings?**

- The average costs (adjusted for total service) of public ambulance companies were higher than the average costs of private ambulance companies.
- Public and private companies had a significantly different distribution of CPT code usage frequencies.

**What are the implications of the main findings?**

- Our findings highlight the differences in ambulance billing practices and underscore how patients are at risk of financial uncertainty.
- This study supports the need for legislative protections and greater cost transparency in ambulance services for patients.

**Abstract:**

**Introduction:** The costs associated with ambulance services are varied and poorly understood, which may contribute to financial burden and barriers to care for patients. **Methods:** We describe differences in ambulance service costs, comparing public versus private companies, by using the Centers for Medicare and Medicaid Services public use files. We determined the two largest public and two largest private ambulance companies in each state and calculated the average miles traveled per ambulance ride, number of trips by company, adjusted cost, and CPT code usage. We compared these variables between 2019 and 2021, across nine geographic divisions of the US. **Results:** In both 2019 and 2021, the average costs (adjusted for total service) of public companies were higher than the average costs of private companies. In both years, public companies had fewer average miles traveled compared to private companies. The distribution of CPT codes used was significantly different in public and private companies. The CPT code used most frequently by public companies was more expensive than the CPT code used most often by private companies. **Conclusions:** Differences in ambulance billing practices may contribute to financial uncertainty for patients. This study underscores the need for further investigation into the factors driving these disparities to inform policy decisions and improve cost transparency for patients.

## 1. Introduction

The costs associated with ambulance services in the U.S. are nuanced, which may contribute to uncertainty and distress for patients and their loved ones when ambulance services are needed. As with many facets of healthcare costs in the U.S., the cost of ground ambulance transportation has consistently increased over the past decade [1]. The average price of a ground ambulance trip in 2021 was over $1000 per ride, which had risen by 33% since 2012 [1]. Additionally, the average out-of-pocket cost for a ground ambulance ride in 2021 was $229, a 64% increase from 2012 [1]. Moreover, even in recent years, ambulance costs have substantially increased, with estimates suggesting the cost of Advanced Life Support (ALS) and Basic Life Support (BLS) ambulance transports increased by 23% and 18% respectively from 2017 to 2020 [2,3]. Thus, further investigation into factors driving these increased costs is warranted.

In addition to the costs of ambulance services, the unpredictable nature of the bills can further complicate patients’ experiences with these services. For example, ambulance services may transport patients to out-of-network hospitals, which can lead to unexpected medical bills [4,5]. From 2013 to 2017, 71% of ground ambulance rides transporting insured patients resulted in surprise out-of-network bills [2]. Notably, the 2018 Kaiser Family Foundation Health Tracking Poll found that two-thirds of U.S. adults were either somewhat or very worried about unexpected medical bills [6,7]. In 2020, the “No Surprises Act” was passed, which aimed to protect patients from unexpected medical bills resulting from the unintentional use of out-of-network providers at in-network facilities; however, this act does not apply to ground ambulance bills [6]. These factors pose a problematic level of uncertainty for patients, yet a dearth of research exists investigating the costs associated with ambulance services, highlighting the need for further investigation into these issues.

With the current study, we sought to describe the landscape of ambulance services in the U.S. Specifically, we aimed to describe the differences in costs, distances traveled, and Current Procedural Terminology (CPT) code usage between the two most utilized public companies and the two most utilized private companies per U.S. state. We also aimed to describe how these variations differ across geographic regions of the U.S. We limited our analysis to the two largest public and private ambulance service providers within each state, because they serve large geographic areas and collectively represent a substantial proportion of ambulance transports within their respective states. Including only the largest providers allowed us to systematically obtain comparable and verifiable pricing information across states while maintaining methodological consistency. We hypothesized that private companies may be costlier than public companies, as private institutions may have more incentive to charge consumers more than federally/state-regulated public companies. By demonstrating discrepancies among the cost and utilization of ambulance services across the U.S., we hope to provide a strong foundation for providing cost transparency of ambulance services to the general population. Understanding these differences may inform efforts aimed at reducing excessive financial burden for patients [7].

## 2. Materials and Methods

We conducted a descriptive analysis of the billing practices of public and private ambulance companies in the U.S. We obtained data from the 2019 and 2021 Centers for Medicare and Medicaid Services (CMS) ambulance services public use files. For both years, we separated the companies by state and ranked them numerically by total beneficiaries, which represents the number of customers served each year by the company. We selected the two public and private companies with the highest number of total beneficiaries in each state. This resulted in a total of 203 companies included in our analysis. We selected our companies based on the 2021 data and used the same companies for the 2019 analysis (we wanted to have a pre-COVID comparison period and thus chose 2019 for this reason). We grouped these companies by ownership structure (public vs. private) and geographic location according to the nine major geographic regions defined by the U.S. Census Bureau.

Each company has associated CPT codes representing the various levels of clinical care that stratify the billing procedures of each ambulance ride. Our study used ground ambulance CPT codes (A0425, A0426, A0427, A0428, A0429, A0433, A0434). Medicare calculates cost per code by multiplying the total relative value units (RVUs) by a conversion factor with geographic adjustments [8,9]. To calculate the data used in our final analysis, we used the total services and average Medicare standardized payment amount for each CPT code per company. Total services represent the number of times the company bills each CPT code in a year. When a company bills a CPT code, the Medicare standardized payment is the amount Medicare pays the company after removing geographic differences in payment rates. Using these items from the CMS public use files and the CPT codes, we calculated the main outcomes of our study: average cost adjusted by total service, average miles driven per ambulance ride, and frequency of CPT code usage.

To estimate the average adjusted cost per company, we calculated an average of the Medicare standardized payment amount for each CPT code mathematically adjusted by the total services value of the code. This created a single value for each company that represents the average amount the company receives from Medicare when billing for services. We did not use the A0425 code in this analysis as it is used to calculate cost per mile and is billed for each mile traveled instead of each ambulance ride. We then compared the average adjusted cost between public and private companies and across geographic divisions in 2019 and 2021. We also compared the average adjusted cost between 2019 and 2021 to capture any change across that time interval.

To calculate the number of miles driven per ambulance ride, we first calculated a sum of the total services of each non-cost per mile CPT code (A0426, A0427, A0428, A0429, A0433, A0434) for each company to produce a value that represents the total number of ambulance rides for that year. We then divided the total services for the cost per mile CPT code (A0425; which is equal to the total number of miles driven by the company for the entire year) by that sum to produce the average miles traveled per ambulance ride. We compared this value between public and private companies in both 2019 and 2021 and between those two years.

To determine the relative frequency of CPT code usage, we calculated a sum of the total services of the non-cost per mile CPT codes (A0426, A0427, A0428, A0429, A0433, A0434) for each company and divided each individual CPT code’s total services by this sum to produce a usage proportion for each CPT code. We then calculated an average of the usage proportions for each code in public and private companies and compared them to one another for 2019 and 2021. We also compared the usage proportion of each code between 2019 and 2021.

### Statistical Analysis

We analyzed the data using descriptive statistics and nonparametric methods. For each NPI/company, we excluded records with code “A0425” (mileage) or missing cost data. We weighted Medicare standardized costs by total services and averaged them within each company, then summarized costs as median [IQR] by group (public vs. private), stratified by geographic division and year. We calculated trips and miles by summing non-mileage and “A0425” codes, respectively, within each company year, and summarized them in the same way. We estimated confidence intervals using bootstrap resampling.

We performed bivariate comparisons using permutation tests based on Wilcoxon rank-sum statistics, clustering at the National Provider Identifier level within states (1000 iterations). We conducted year-to-year comparisons (2021 vs. 2019) for costs, trips, and miles using paired Wilcoxon signed-rank tests on within-company differences. We evaluated CPT code usage using permutation tests based on chi-square statistics, permuting year within company to preserve total code volume and stratifying by type (one-sided tests). We applied Bonferroni correction for multiple testing (2 strata for outcome-level analyses; k = 18 for regional analyses). We defined statistical significance as *p* < 0.05.

## 3. Results

### 3.1. Adjusted Cost by Total Service

**Figure 1:**
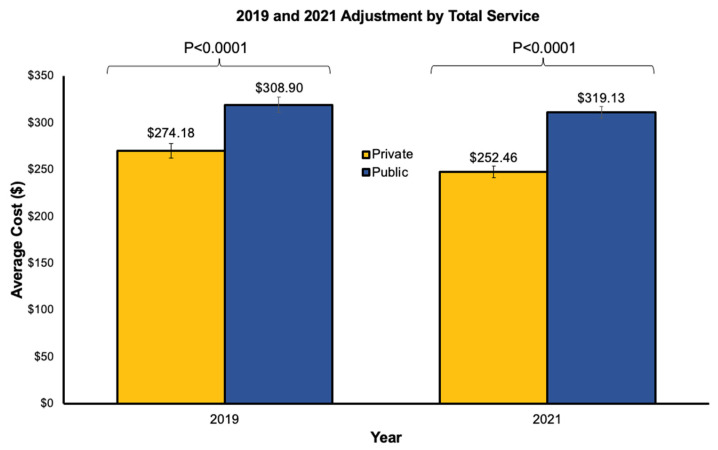
Average ambulance costs adjusted by total service in 2019 and 2021. Average cost adjusted by total service for top 2 private and top 2 public ambulance companies in 2019 and 2021.

For 2019, adjusted cost by total service for the top two public services was higher than private services ($308.90 [289.25, 328.86] vs. $274.18 [231.71, 310.92], *p* < 0.0001) (Figure 1). Public companies were higher than private companies in average cost adjusted by total service across the New England and South Atlantic geographic divisions (Table 1). The range of public adjusted cost by geographic division was $294.12 [279.04, 314.28] (East North Central) to $322.45 [291.80, 330.31] (Pacific). The range of private adjusted cost by geographic division was $229.67 [211.48, 266.70] (East North Central) to $333.76 [298.74, 435.36] (Middle Atlantic).

In 2021, regarding the average cost adjusted by total services provided, the top two public companies were higher than the top two private companies ($319.13 [299.32, 332.70] vs. $252.46 [197.38, 297.09], *p* < 0.0001, Figure 1). The differences in cost adjusted by total service were also apparent across the East North Central and South Atlantic geographic divisions (Table 1). The range of public adjusted cost by geographic division was 306.99 [300.52, 319.93] (Middle Atlantic) to $333.53 [318.66, 342.17] (Pacific). The range of private adjusted cost by geographic division was $194.25 [177.88, 246.38] (South Atlantic) to $301.57 [237.29, 319.69] (West North Central).

When comparing 2019 and 2021, we found there was no difference between average adjusted cost for public companies ($308.90 [289.25, 328.86] vs. $319.13 [299.32, 332.70], *p* = 0.0517), but private companies had higher average adjusted cost in 2019 than in 2021 ($274.18 [231.71, 310.92] vs. $252.46 [197.38, 297.09], *p* = 0.0008).

### 3.2. Distance and Utilization

**Figure 2:**
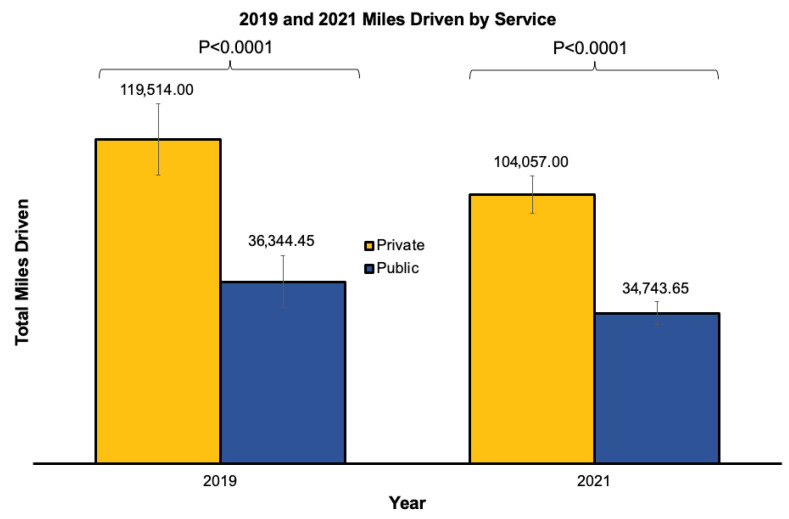
Average total miles driven per service in 2019 and 2021. Averaged total miles driven per total ground ambulance service comparing top two private and top two public companies for 2019 and 2021 in the United States.

In 2019, public companies made an average of 4449.00 [1703.50, 10,202.25] trips whereas private companies made an average of 5632.50 [1578.25, 17,169.00] trips (*p* = 1.000) (Table 2). The top two public companies drove fewer miles than the top two private companies (36,344.45 [18,278.60, 85,117.57] miles vs. 119,514.00 [49,449.53, 265,784.50] miles, *p* < 0.0001, Table 3, Figure 2) overall and in the New England, East North Central, and East South Central geographic regions.

In 2021, public companies made fewer trips than private companies (5633.00 [2405.50, 11,737.25] trips vs. 8980.00 [3385.00, 16,431.00] trips, *p* = 0.0180, Table 2) overall and in all geographic regions except West North Central, South Atlantic, Mountain, and Pacific. The top two public ambulance companies drove fewer miles when compared to the top two private ambulance companies (34,743.65 [18,261.93, 84,694.32] miles vs. 104,057.00 [38,802.10, 194,833.60] miles, *p* < 0.0001, Table 3, Figure 2) overall and in the New England geographic region.

When comparing 2019 and 2021, both public and private companies traveled more miles in 2019 than in 2021 (36,344.45 [18,278.60, 85,117.57] miles vs. 34,743.65 [18,261.93, 84,694.32] miles, *p* < 0.0001; 119,514.00 [49,449.53, 265,784.50] miles vs. 104,057.00 [38,802.10, 194,833.60] miles, *p* = 0.0019).

### 3.3. CPT Code Usage

**Figure 3:**
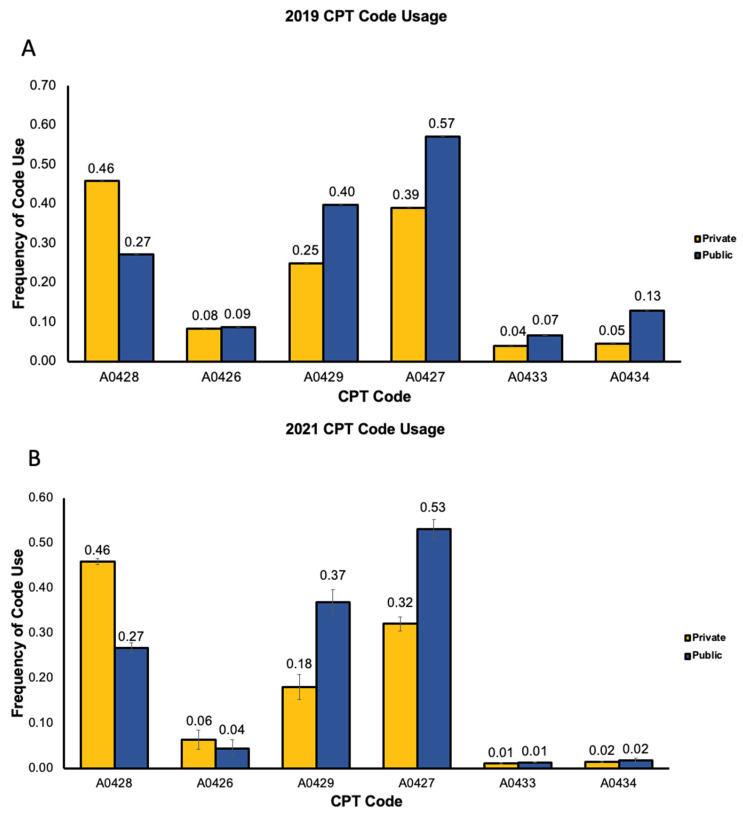
Frequency of CPT code usage in 2019 and 2021. (**A**). Frequency of CPT code usage for top two private and top public companies in 2019. (**B**). Frequency of CPT code usage for top two private and top public companies in 2021. A0428: basic life support, non-emergency transport; A0426: advanced life support, non-emergency transport, Level 1; A0429: basic life support, emergency transport; A0427: advanced life support, emergency transport, Level 1; A0433: advanced life support, Level 2; A0434: specialty care transport.

In 2019, public and private companies overall had a significantly different CPT code usage distribution (*p* < 0.0001). Public companies charged for A0428 (basic life support, non-emergency transport) on average less often than private companies (0.40 vs. 0.46, Figure 3A). Public companies charged for A0427 (advanced life support, non-emergency transport, Level 1), A0429 (basic life support, emergency transport), and A0434 (specialty care transport) on average more often than private companies (0.57 vs. 0.39, 0.40 vs. 0.25, and 0.13 vs. 0.05 respectively, Figure 3A). The most commonly used CPT code for public companies was A0427 at a frequency of 0.57 and for private companies was A0428 at a frequency of 0.46 (Figure 3A). The CPT code used on average the least often for public and private companies was A0433 (advanced life support, Level 2) at a frequency of 0.04 and 0.07, respectively (Figure 3A).

In 2021, public and private companies distributed their CPT codes differently overall (*p* < 0.0001). Private companies charged for A0428 on average more often than public companies (0.46 vs. 0.27, Figure 3B). Public companies charged for A0427, A0429, and A0433 on average more often than private companies (0.53 vs. 0.32, 0.37 vs. 0.18, and 0.01 vs. 0.01 respectively, Figure 3B). The most used CPT code for public companies was A0427 at a frequency of 0.53 (Figure 3B) and for private companies was A0428 at a frequency of 0.46. The CPT code used on average the least often for public and private companies was A0433 at a frequency of 0.01 and 0.01, respectively (Figure 3B).

When comparing 2019 and 2021, the distribution of CPT code usage for private companies was significantly different in 2019 than in 2021 (*p* < 0.0001); however, it was only marginally different for public companies (*p* = 0.0640).

## 4. Discussion

In this study, we demonstrated significant cost discrepancies between public and private ambulance service companies. Specifically, we found that in both 2019 and 2021, public companies had significantly higher costs adjusted by total service than private companies overall and in the majority (six of nine) of geographic regions, and public companies used higher-cost CPT codes more often than private companies. Our findings suggest that public ambulance services appear generally more expensive than private ambulance services in this dataset. However, further research is needed to understand the nuanced factors that may explain this finding. Collectively, these results support the overall concept that ambulance services in the U.S. are poorly understood and exhibit a problematic lack of transparency [2,10,11,12].

Our work underscores the complexity of costs associated with ambulance services. Prior work has described ambulance service costs increasing over time, but no prior research had compared public and private companies, nor the factors associated with their differences [13]. In our study, we found the public and private cost discrepancy may be associated with differences in CPT coding patterns. For example, we evaluated six CPT codes used for ground ambulance services and certain CPT codes representing higher levels of care correlate with higher costs. Our data underscore that public and private companies have a significantly different distribution of CPT code billing. Due to limitations in our methodology, we cannot determine the factors underlying this finding; thus, further investigation into CPT code usage is needed to identify why public companies might charge more than private companies. We hope to encourage future research aimed at understanding how these disparities in costs are implemented, and to develop interventions to establish equity among billing patients for ambulance services.

Notably, we also found discrepancies in distances traveled for public versus private ambulance companies. In both 2019 and 2021, public companies traveled significantly fewer miles than private companies. Thus, although public companies charge a higher cost, our findings suggest that they travel shorter distances than private companies. This paradox may reflect the possibility that public ambulance services transporting patients within a closer proximity to hospitals, such as in urban communities, as response times are significantly shorter in urban areas than rural areas [14,15,16]. In contrast, private ambulance services may operate more frequently in rural regions where healthcare utilization is lower and patients often seek care only for more severe conditions, which may necessitate transport over longer distances to reach appropriate facilities [17,18,19,20,21]. However, further investigation is needed to definitively identify the factors behind the miles traveled discrepancy between public and private companies.

By comparing datasets from 2019 and 2021, we aimed to demonstrate how the COVID-19 pandemic may have influenced ambulance service utilization. We found the adjusted costs for total services in 2019 were higher than the adjusted costs for total services in 2021, but only in private companies. Additionally, private companies had a significant difference in CPT code billing patterns between 2019 and 2021. Plausibly, mortality displacement could have played a role, whereby higher mortality among high-risk individuals early in the pandemic may have resulted in fewer severely ill patients later in the study period, resulting in fewer advanced CPT codes. However, with the limitations of our current study, we cannot definitively attribute our findings to the notion of mortality displacement [22,23,24,25,26]. Therefore, additional research is needed to assess how COVID-19 may have affected ambulance cost and CPT code use.

Our study has several limitations to note. First, our analyses relied on a restricted sample of only four companies per state rather than all providers nationwide, which may limit the national generalizability of our findings. Second, our use of Medicare standardized payments to represent ambulance service costs does not account for negotiated rates or direct patient charges, which limits our ability to estimate patient out-of-pocket costs. Third, the CMS ambulance datasets do not include if private ambulance companies are non-profit or for-profit, which may result in public ambulance companies seemingly charging more for their services in comparison to non-profit organizations. Next, the dataset lacks information about the area of coverage for a particular service, including population density, which could result in discrepancies in distance traveled by ambulance companies per ride [27]. Additionally, factors influencing the dispatchers’ choice on sending public versus private companies to transport patients is largely variable among circumstance [4]. This leaves an unresolved question as to whether public companies serve higher acuity patients more often than private companies. Future research should seek to determine the true severity of patients’ conditions based on the disposition of the patient (i.e., hospitalized, death, discharged from the emergency room) cross-examined with the CPT code billing of their ambulance rides to further understand the cost discrepancies found in our study. Lastly, we lack information about patients’ sociodemographic factors and their clinical outcomes from this study, thus we were unable to stratify analyses according to these variables. Future work is needed to understand how factors not captured in this study correlate with ambulance costs and use.

## 5. Conclusions

In conclusion, our study provides novel findings to help characterize the complexity of ambulance services in the U.S. Considering ground ambulance services remain excluded from the No Surprises Act, this study further supports that current federal protections may be insufficient to protect patients [6]. Our observed discrepancies in billing practices across geographic regions and public versus private ownership highlight the need for greater transparency in emergency medical services billing. Our work may help inform future efforts to improve cost transparency and consideration of regulatory oversight for ambulance services.

## Figures and Tables

**Table 1:** Adjusted cost by geographic regions in 2019 and 2021.

**Geographic Division** **for 2019**	**Private** **Adjusted** **Cost ($)**	**Public** **Adjusted Cost ($)**	***p*** **-Value**
Overall	274.18 [231.71, 310.92]	308.90 [289.25, 328.86]	<0.0001
New England	248.41 [221.70, 267.26]	316.08 [292.70, 321.03]	<0.0001
Middle Atlantic	333.76 [298.74, 435.36]	297.71 [291.95, 349.89]	1.0000
East North Central	229.67 [211.48, 266.70]	294.12 [279.04, 314.28]	0.882
West North Central	286.11 [243.56, 306.54]	298.60 [291.80, 319.89]	1.0000
South Atlantic	235.51 [191.52, 269.44]	317.77 [297.68, 331.36]	<0.0001
East South Central	271.67 [241.48, 282.52]	304.24 [281.95, 325.55]	1.0000
West South Central	262.66 [244.83, 333.39]	321.66 [287.32, 331.87]	1.0000
Mountain	295.73 [289.50, 312.37]	310.41 [295.12, 327.15]	1.0000
Pacific	290.84 [245.47, 384.49]	322.45 [291.80, 330.31]	1.0000
**Geographic Division** **for 2021**	**Private** **Adjusted** **Cost ($)**	**Public** **Adjusted Cost ($)**	***p*** **-value**
Overall 2021	252.46 [197.38, 297.09]	319.13 [299.32, 332.70]	<0.0001
New England	212.66 [187.99, 255.15]	323.21 [312.73, 327.22]	0.1440
Middle Atlantic	244.38 [211.43, 297.26]	306.99 [300.52, 319.93]	1.0000
East North Central	219.05 [191.45, 229.58]	313.78 [277.34, 335.07]	<0.0001
West North Central	301.57 [237.29, 319.69]	310.21 [301.71, 324.65]	1.0000
South Atlantic	194.25 [177.88, 246.38]	324.83 [297.40, 335.74]	<0.0001
East South Central	266.24 [248.83, 281.67]	324.89 [285.97, 332.26]	1.0000
West South Central	250.47 [233.97, 281.33]	317.02 [299.04, 322.29]	0.0900
Mountain	299.34 [248.00, 306.48]	310.81 [306.86, 329.14]	1.0000
Pacific	284.70 [247.94, 326.89]	333.53 [318.66, 342.17]	1.0000

Average cost adjusted by total service for top 2 private and top 2 public ambulance companies in 2019 and 2021 organized by geographic division as described by the CDC.

**Table 2:** Average number of trips traveled by geographic regions in 2019 and 2021.

**Geographic Division** **for 2019**	**Private** **Trips by Company**	**Public** **Trips by Company**	***p*** **-Value **
Overall	5632.50 [1578.25, 17,169.00]	4449.00 [1703.50, 10,202.25]	1.0000
New England	6898.00 [2122.50, 30,522.50]	2184.00 [1728.00, 3515.50]	<0.0001
Middle Atlantic	1416.00 [583.00, 18,943.00]	3151.00 [1613.00, 4708.75]	1.0000
East North Central	13,969.00 [7212.50, 34,308.25]	7532.00 [2273.00, 11,280.50]	<0.0001
West North Central	1907.50 [759.50, 16,652.25]	6048.30 [1030.25, 9944.00]	<0.0001
South Atlantic	10,116.00 [2929.00, 18,064.00]	5924.50 [2604.50, 19,188.00]	<0.0001
East South Central	17,017.50 [13,235.75, 20,851.50]	6375.50 [4959.00, 8851.25]	<0.0001
West South Central	11,115.00 [6989.00, 33,735.00]	6467.00 [4343.00, 21,700.00]	<0.0001
Mountain	2827.00 [1278.50, 4825.00]	1492.00 [551.00, 4719.00]	1.0000
Pacific	1969.00 [395.00, 6957.00]	2689.00 [2064.00, 15,923.00]	1.0000
**Geographic Division** **for 2021**	**Private** **Trips by Company**	**Public** **Trips by Company**	***p*** **-value **
Overall 2021	8980.00 [3385.00, 16,431.00]	5633.00 [2405.50, 11,737.25]	0.0180
New England	5660.00 [3917.00, 15,026.00]	1979.00 [1631.50, 2658.25]	<0.0001
Middle Atlantic	19,315.00 [14,194.00, 36,770.00]	5155.50 [3584.50, 10,285.25]	<0.0001
East North Central	16,542.00 [10,862.25, 39,084.50]	7195.50 [5345.25, 9186.00]	<0.0001
West North Central	3653.50 [1761.25, 9557.50]	5520.50 [1789.50, 8257.00]	1.0000
South Atlantic	13,154.00 [7034.00, 15,671.00]	11,895.50 [7526.50, 21,184.00]	1.0000
East South Central	16,622.00 [13,041.00, 21,582.25]	6621.50 [5220.00, 7996.00]	<0.0001
West South Central	12,567.00 [8512.00, 35,494.00]	17,193.00 [5344.00, 18,814.00]	<0.0001
Mountain	3385.00 [1369.50, 7526.00]	3360.00 [1386.25, 5982.00]	1.0000
Pacific	5964.50 [2905.25, 12,560.50]	3418.00 [2437.00, 13,940.00]	0.1800

Averaged number of trips taken for top 2 private and top 2 public ambulance companies in 2019 and 2021 organized by geographic division as described by the CDC.

**Table 3:** Average total miles traveled by geographic regions in 2019 and 2021.

**Geographic Division** **for 2019**	**Private** **Miles by Company**	**Public** **Miles by Company**	***p*** **-Value **
Overall	119,514.00 [49,449.53, 265,784.50]	36,344.45 [18,278.60, 85,117.57]	<0.0001
New England	158,864.60 [85,621.35, 345,370.95]	5951.00 [5337.25, 23,344.00]	<0.0001
Middle Atlantic	141,384.10 [128,315.80, 259,554.90]	40,769.65 [33,252.83, 48,572.35]	1.0000
East North Central	306,602.50 [171,816.50, 397,905.40]	61,037.40 [35,589.62, 71,819.48]	0.0360
West North Central	78,583.80 [33,010.20, 271,468.75]	59,851.30 [29,201.50, 84,227.10]	1.0000
South Atlantic	148,372.20 [58,912.13, 250,214.65]	91,101.60 [37,474.90, 138,288.40]	1.0000
East South Central	205,652.25 [64,207.23, 421,920.90]	50,895.55 [22,865.75, 75,153.67]	<0.0001
West South Central	277,975.15 [221,338.67, 678,030.95]	124,958.20 [45,527.15, 151,753.00]	1.0000
Mountain	27,651.50 [18,096.53, 81,612.48]	21,811.45 [13,939.40, 29,711.10]	1.0000
Pacific	83,215.00 [64,156.20, 103,002.88]	67,491.80 [20,450.10, 115,315.60]	1.0000
**Geographic Division** **for 2021**	**Private** **Miles by Company**	**Public** **Miles by Company**	***p*** **-value **
Overall 2021	104,057.00 [38,802.10, 194,833.60]	34,743.65 [18,261.93, 84,694.32]	<0.0001
New England	70,869.40 [49,095.90, 143,499.80]	6844.50 [4917.80, 19,280.53]	<0.0001
Middle Atlantic	122,214.60 [105,373.00, 183,012.80]	39,227.45 [34,461.20, 43,330.25]	0.1980
East North Central	226,398.80 [140,085.05, 398,560.78]	49,214.90 [28,079.63, 79,220.45]	0.0720
West North Central	46,970.40 [31,129.72, 148,878.05]	26,522.25 [21,751.42, 70,155.57]	1.0000
South Atlantic	178,701.80 [109,873.90, 205,998.20]	87,643.95 [49,527.20, 118,189.72]	0.0540
East South Central	217,252.40 [124,823.53, 349,506.58]	50,384.65 [23,793.85, 67,575.58]	0.0720
West South Central	258,121.60 [160,068.30, 619,291.70]	103,828.20 [44,122.10, 152,962.50]	0.4680
Mountain	25,310.60 [16,986.20, 56,965.90]	20,207.00 [12,817.07, 31,992.87]	1.0000
Pacific	47,379.85 [33,384.20, 89,391.88]	24,236.90 [18,577.00, 103,805.60]	1.0000

Averaged total miles driven for top 2 private and top 2 public ambulance companies in 2019 and 2021 organized by geographic division as described by the CDC.

## Data Availability

Data presented in this manuscript is publicly available online at the “Centers for Medicare and Medicaid Services (CMS) ambulance services public use files” (https://www.cms.gov/medicare/payment/fee-schedules/ambulance/ambulance-fee-schedule-public-use-files, accessed on 11 January 2024).

## Abbreviations

The following abbreviations are used in this manuscript:

CPT	Current Procedural Terminology
CMS	Centers for Medicare and Medicaid Services
